# A Rare Case of Gastric Outlet Obstruction With Severe Reflux Esophagitis Due to a Percutaneous Endoscopic Gastrostomy Tube Balloon Displacement

**DOI:** 10.7759/cureus.18635

**Published:** 2021-10-09

**Authors:** Adham E Obeidat, Ratib Mahfouz, Mohammad R Darweesh, Herbert Lim

**Affiliations:** 1 Internal Medicine, University of Hawaii, Honolulu, USA; 2 Internal Medicine, Kent Hospital, Warwick, USA; 3 Internal Medicine, East Tennessee State University, Johnson City, USA; 4 Gastroenterology and Hepatology, The Queen's Medical Center, Honolulu, USA

**Keywords:** esophagitis, dysphagia, gi bleeding, gastric outlet obstruction, peg tube

## Abstract

In patients with a functional gastrointestinal (GI) tract, enteral feeding is preferred over parenteral feeding as it has fewer complications and a relatively lower cost. Nasogastric and nasoenteric feeding tubes are available options but when long-term enteral feeding is desired, a percutaneous endoscopic gastrostomy (PEG) tube is more convenient. PEG tube can be associated with multiple complications; however, its displacement which causes gastric outlet obstruction (GOO) is a rare one. Here we present a case of an 81-year-old woman with dementia who presented with upper GI bleeding and was found to have GOO causing reflux esophagitis due to PEG tube displacement.

## Introduction

Inability to obtain physiological enteral access usually necessitates looking for alternative methods for feeding. Enteral feeding is preferred over parenteral feeding in patients with a functional gastrointestinal (GI) tract as it has fewer complications, lower cost, and the ability to decrease the risk of bacterial translocation and infection [1-3]. Temporary access can be achieved by a nasogastric or a nasoenteric feeding tube, and despite their ease of insertion, they are prone to dislodgement and clogging when used for a long time [4]. Therefore, the percutaneous endoscopic gastrostomy (PEG) tube is more convenient when long-term enteral feeding is desired.

PEG tubes were first introduced in 1980 by Ponsky and Gauderer [5,6]. Insertion by endoscopy is the preferred method due to its low cost, lower invasiveness, and the lack of need for general anesthesia [7]. It is often placed in patients with prolonged reduced levels of consciousness, dementia, Parkinson’s disease, obstructive oropharyngeal or esophageal malignancies, and gastric outlet obstruction (GOO) [8]. PEG tubes can be associated with multiple complications that range from wound infection and bleeding to more serious complications such as fistula formation, necrotizing fasciitis, and perforation [9]. However, a PEG tube displacement causing GOO is a rare complication. Here we present a case of an 81-year-old woman with dementia who presented with upper GI bleeding and was found to have GOO causing reflux esophagitis due to the PEG tube displacement.

This case will be presented as a poster in the American College of Gastroenterology (ACG) Annual Meeting 2021, Las Vegas, Nevada, USA.

## Case presentation

An 81-year-old woman with a past medical history of hypothyroidism and Parkinson's disease complicated by dementia and dysphagia, with a feeding PEG tube that was placed six years prior to presentation, presented to the emergency room (ER) with hematemesis and melena. Her symptoms started one day prior to admission. The patient was also complaining of abdominal pain, however, there was no associated dysphagia, change in bowel habits, or bleeding from other sites. She did not have a history of GI bleeding nor was she taking aspirin or anti-coagulants.

In the ER, the patient was tachycardic and hypotensive, but afebrile. Her complete blood count (CBC) was significant for a white blood cell (WBC) count of 12.17 x 10(3)/uL, a slightly dropped hemoglobin of 10.4 g/dL from her baseline of 11.6 g/dL, and a normal platelet count. The basic metabolic panel was only significant for hypocalcemia of 5.5 mg/dL with serum albumin of 2.2 gm/dL. Liver function test, partial thromboplastin time (PTT), prothrombin time (PT), and international normalized ratio (INR) were all normal. She was given three units of packed red blood cells and was started on a proton pump inhibitor (PPI), but given the recurrent hematemesis and decreased level of consciousness, she was intubated for airway protection and was placed on mechanical ventilation.

**Figure 1:**
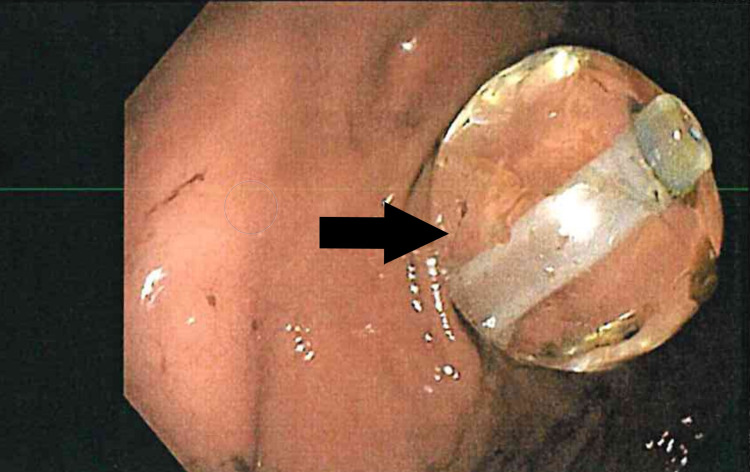
Endoscopic image shows a displaced PEG tube balloon causing obstruction of the gastric outlet. PEG, percutaneous endoscopic gastrostomy.

Esophagogastroduodenoscopy (EGD) was done, which showed Los Angeles (LA) grade D esophagitis and a non-bleeding gastric ulcer. Moreover, the PEG tube balloon tip was displaced and trapped in the duodenal bulb causing GOO (Figure 1) with resultant severe reflux esophagitis. The balloon tip was retracted to the gastric wall and fixed. The patient was continued on a PPI and was extubated the day after. Tube feeding was resumed, and the patient was discharged on a daily PPI.

## Discussion

Neurological disorders that affect swallowing may require alternative means to provide enteral nutrition. Patients who need enteral nutrition for longer than four to six weeks can benefit from PEG tubes as they are a simple and relatively low-cost way to provide nutrition [10]. Multiple methods of placement are available; the percutaneous approach is more common as it is faster and more cost-effective when compared to the surgical approach. However, there is no difference in morbidity or mortality between the two approaches [11]. Our patient had Parkinson’s disease, which affects her ability to swallow food, and she had a feeding gastrostomy tube that was placed percutaneously.

Complications of PEG tubes can range from minor complications such as wound infection and minimal bleeding to major complications such as fistulas and necrotizing fasciitis [9]. The rate of complications varies between 16% and 70%, while the most common reported complication is tube dislodgement [9]. Age and comorbidities increase the risk of complications [12]. Our patient is elderly and she has multiple comorbidities, which put her at an increased risk for complications.

Complications can be early, late, or not related to time [9,12]. These include tube dysfunction such as clogging, tube deterioration, and early balloon deflation. Moreover, infections such as wound infection and necrotizing fasciitis can occur at any time and they need to be treated depending on the response to antibiotics and the viability of the infected tissue. Indications of tube removal are peritoneal signs of necrotizing fasciitis [9]. Povidone-iodine and prophylactic antibiotics are important to lower the risk of wound infection [13,14]. Bleeding is a rare complication and most of the time it can be controlled with simple pressure. On the other hand, ulceration and peristomal leak are common side effects [9].

Occasionally, PEG tubes may migrate into the duodenum, obstructing the gastric outlet [15]. This can happen when the external balloon/bolster slides away from the abdominal wall, which allows the tube to slide into the stoma tract, bypassing the pylorus and residing in the duodenum and thus leading to obstruction [16,17]. Careful preparation, placement, and after-care are important to avoid such complications. To prevent internal sliding of the tube, Haynes et al. recommended affixing the external bolster to the tube with a silk suture [18]. Our patient was found to have a dislodged tube causing GOO, resulting in severe esophagitis with hematemesis and melena.

## Conclusions

The PEG tube is the preferred method to provide prolonged enteral feeding in patients with dementia, neurological disorders, and dysphagia. However, PEG tubes can be associated with multiple complications including tube dislodgment, which may cause GOO, and in rare cases, such as our case, it may lead to severe reflux esophagitis and bleeding. Therefore, clinicians should be aware of possible complications of PEG tubes, including GOO, and upper GI bleeding when dealing with such patients.
